# Effect of Graded Nrf2 Activation on Phase-I and -II Drug Metabolizing Enzymes and Transporters in Mouse Liver

**DOI:** 10.1371/journal.pone.0039006

**Published:** 2012-07-12

**Authors:** Kai Connie Wu, Julia Yue Cui, Curtis D. Klaassen

**Affiliations:** Department of Pharmacology, Toxicology, and Therapeutics, University of Kansas Medical Center, Kansas City, Kansas, United States of America; University Paris Diderot-Paris 7, France

## Abstract

Nuclear factor erythroid 2-related factor 2 (Nrf2) is a transcription factor that induces a battery of cytoprotective genes in response to oxidative/electrophilic stress. Kelch-like ECH associating protein 1 (Keap1) sequesters Nrf2 in the cytosol. The purpose of this study was to investigate the role of Nrf2 in regulating the mRNA of genes encoding drug metabolizing enzymes and xenobiotic transporters. Microarray analysis was performed in livers of Nrf2-null, wild-type, Keap1-knockdown mice with increased Nrf2 activation, and Keap1-hepatocyte knockout mice with maximum Nrf2 activation. In general, Nrf2 did not have a marked effect on uptake transporters, but the mRNAs of organic anion transporting polypeptide 1a1, sodium taurocholate cotransporting polypeptide, and organic anion transporter 2 were decreased with Nrf2 activation. The effect of Nrf2 on cytochrome P450 (Cyp) genes was minimal, with only Cyp2a5, Cyp2c50, Cyp2c54, and Cyp2g1 increased, and Cyp2u1 decreased with enhanced Nrf2 activation. However, Nrf2 increased mRNA of many other phase-I enzymes, such as aldo-keto reductases, carbonyl reductases, and aldehyde dehydrogenase 1. Many genes involved in phase-II drug metabolism were induced by Nrf2, including glutathione *S*-transferases, UDP- glucuronosyltransferases, and UDP-glucuronic acid synthesis enzymes. Efflux transporters, such as multidrug resistance-associated proteins, breast cancer resistant protein, as well as ATP-binding cassette g5 and g8 were induced by Nrf2. In conclusion, Nrf2 markedly alters hepatic mRNA of a large number of drug metabolizing enzymes and xenobiotic transporters, and thus Nrf2 plays a central role in xenobiotic metabolism and detoxification.

## Introduction

Nuclear factor erythroid 2-related factor 2 (Nrf2) is a transcription factor that induces a battery of cytoprotective genes in response to oxidative/electrophilic stress. Under physiological conditions, Nrf2 is bound to its repressor Kelch-like ECH associating protein 1 (Keap1) in the cytosol. Keap1 functions as an adapter protein that retains Nrf2 in the cytoplasm by interacting with the cytoskeleton, and it facilitates degradation of Nrf2 by binding with Cullin 3-based E3 ligase, a protein complex that ubiquitinates Nrf2 protein for degradation by proteasomes [1]. Upon oxidative/electrophilic stimuli, multiple amino acid residues of the Keap1 protein are modified, which leads to a conformational change of the Keap1 protein structure and disruption of Keap1-Nrf2 binding [2]. Once released from Keap1, Nrf2 translocates into the nucleus, heterodimerizes with small musculo-aponeurotic fibrosarcoma proteins, binds to antioxidant response elements (ARE) in the promoter of numerous genes, and promotes transcription of target genes [3].

The role of Nrf2 in protecting against oxidative and electrophilic stress has been well established, and the majority of genes involved in antioxidant defense have been identified as Nrf2 target genes in various models. For example, genes that are involved in direct reduction of reactive oxygen species (ROS), including superoxide dismutase, catalase, and glutathione peroxidases are induced by Nrf2 [4]. Genes involved in reduction of oxidized proteins, such as thioredoxin-1, thioredoxin reductase-1, and sulfuredoxin, are also Nrf2-target genes [5]. Genes encoding enzymes that synthesize glutathione (GSH), the most abundant cellular thiol resource, namely γ-glutamate-cysteine ligase catalyze subunit (Gclc) and the modifier subunit (Gclm), as well as glutathione synthase (Gss), are known to be Nrf2 target genes. In addition, genes involved in generation of NADPH, the co-substrate to reduce oxidized GSH, such as glucose-6-phosphate dehydrogenase (G6pd) and malic enzyme (Me1), are induced upon Nrf2 activation [5], [6].

Advanced techniques have recently increased the ability to investigate the Keap1-Nrf2 pathway. Chromatin-immunoprecipitation of Nrf2 binding sites with parallel sequencing (ChIP-seq) comparing mouse embryonic fibroblasts (MEF) of Nrf2-null mice and wild-type mice indicated that Nrf2 also plays a role in basal expression of genes involved in cell proliferation [7]. Proteomic analysis of proteins expressed in livers of Nrf2-null mice and wild-type mice revealed decreases in proteins involved in lipid metabolism [8]. Thus, the Keap1-Nrf2 pathway is not limited to antioxidant defense, but is shown to be a central regulator of multiple biological processes.

There is increasing recognition of the role of Nrf2 in regulating drug metabolizing enzymes as well as uptake and efflux transporters, which alter the kinetics and disposition of xenobiotics [9]. For example, Nrf2 has been shown to increase the mRNA of phase-I drug-metabolizing genes, such as NAD(P)H quinone oxidoreductase 1 (Nqo1) and Cyp2a5 [10], as well as phase-II drug-metabolizing enzymes, such as glutathione *S*-transferase and UDP-glucuronosyltransferase [11]. In addition, efflux transporters, such as Mrp3 and Mrp4, can be induced by Nrf2 [12]. Nrf2 deficiency results in decreased, whereas Nrf2 activation results in increased elimination of acetaminophen from the liver [13], and Nrf2 activation increased biliary excretion of sulfobromophthalein by inducing glutathione-*S*-transferase activity [14].

**Figure 1:**
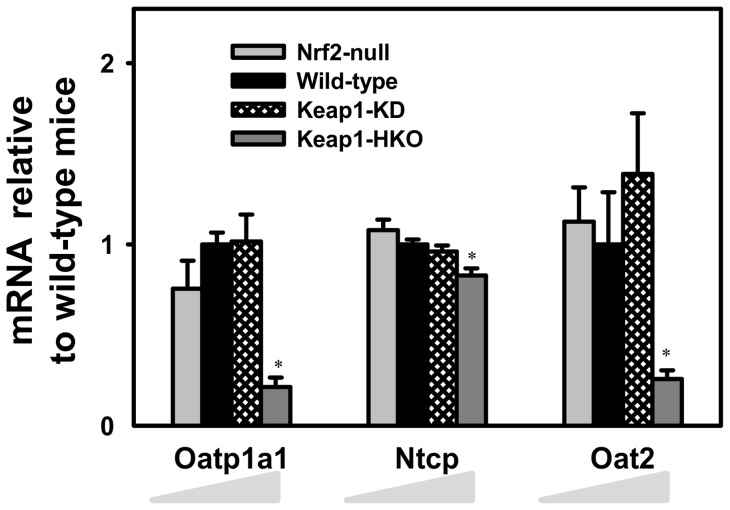
Messenger RNA expression of uptake transporters in a “gene dose-response” model. Data of Nrf2-null, Keap1-KD, and Keap1-HKO mice are normalized by the value of wild-type mice and presented as Mean ± S.E.M. of three mice per group. Asterisks (*) indicate statistically significant differences from wild-type mice (p<0.05).

Liver is the primary organ in the metabolism and detoxification of drugs and environmental toxicants. Whereas Nrf2 is known to regulate a few drug metabolizing enzymes and xenobiotic transporters, there has not been a systematic study to determine the role of Nrf2 in regulating all drug metabolizing enzymes and xenobiotic transporters in liver. In the present study, a “gene dose-response” model with graded hepatic Nrf2 activation was generated by using Nrf2-null, wild-type, Keap1-knockdown (Keap1-KD) mice with enhanced Nrf2 activation in all tissues, and Keap1-hepatocyte knockout (Keap1-HKO) mice with maximum Nrf2 activation in liver. Microarray analyses of livers of the “gene dose-response” model were performed to obtain genomic gene expression profiles to determine the role of Nrf2 in regulating each individual drug metabolizing enzyme and xenobiotic transporter.

**Figure 2:**
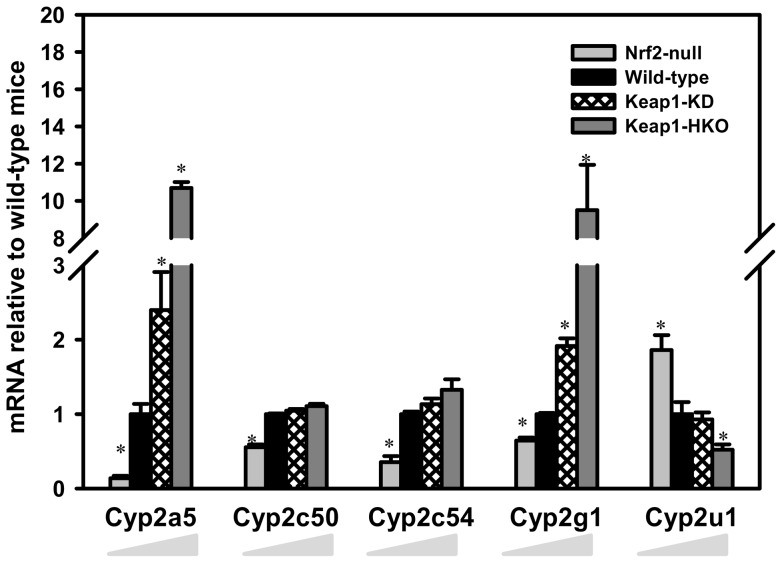
Messenger RNA expression of Cytochrome P450 phase-I drug metabolism enzymes in a “gene dose-response” model. Data of Nrf2-null, Keap1-KD, and Keap1-HKO mice are normalized by the value of wild-type mice and presented as Mean ± S.E.M. of three mice per group. Asterisks (*) indicate statistically significant differences from wild-type mice (p<0.05).

## Methods

### Reagents

All chemicals, unless otherwise specified, were purchased from Sigma-Aldrich (St Louis, MO).

### Ethics statement

Mice were housed according to guidelines of the Institutional Animal Care and Use Committee at the University of Kansas Medical Center, and procedures were carried out in compliance with standards for use of laboratory animals. Animal experiments performed in this manuscript have been approved by the Institutional Animal Care and Use Committee at the University of Kansas Medical Center (protocols 2007–1704 and 2011–1969).

### Animals and husbandry

Eight-week-old C57BL/6 male mice were used for this study. Nrf2-null mice were obtained from Dr Jefferson Chan (University of California, Irvine, CA) [15]. Keap1-KD mice were supplied by Dr Masayuki Yamamoto (Tohoku University, Sendai, Japan). In an attempt to make hepatocyte-specific Keap1-null mice, utilizing a loxP Alb-Cre system, Keap1-KD mice, in which Keap1 was decreased throughout the body, were engineered [16]. Nrf2-null and Keap1-KD mice were backcrossed into the C57BL/6 background, and >99% congenicity was confirmed by the speed congenics group at Jackson Laboratories (Bar Harbor, ME). Keap1-HKO mice were generated by crossing Keap1-KD mice and AlbCre^+^ mice which express Cre only in hepatocytes [5]. Animals were housed in a temperature-, light-, and humidity-controlled environment and had access to Teklad Rodent Diet #8604 (Harlan Laboratories, Madison, WI) and water *ad libitum*. The housing facility is an American Animal Associations Laboratory Animal Care-accredited facility at the University of Kansas Medical Center, and all procedures were approved in accordance with the Institutional Animal Care and Use Committee guidelines.

### Total RNA isolation

Total RNA was isolated using RNAzol B reagent (Tel Test, Inc., Friendswood, TX) according to the manufacturer's protocol. The concentration of total RNA in each sample was quantified spectrophotometrically at 260 nm. The integrity of each RNA sample was evaluated by formaldehyde-agarose gel electrophoresis before analysis.

### Microarray and data analysis

Gene expression in livers of Nrf2-null, WT, Keap1-KD, and Keap1-HKO mice was determined using Affymetrix Mouse 430.20 arrays by the KUMC Microarray Core Facility. Biological cRNA replicates (n = 3) of each genotype were hybridized to an individual array. Raw data CEL files were imported into the “R” program using the “affy” package, normalized by the Robust Multichip Averaging (gcRMA) package [17], and log2 transformed. The probes with intensities higher than log_2_100 in at least one group were selected for further analysis [18]. Gene annotations were obtained using GeneSpring (Agilent Technologies, Santa Clara, CA), and gene symbols were obtained from the mouse 4302 package.

### Quantification of CAR mRNA expression by RT-PCR assay

Total RNA in mouse livers was reverse transcribed into complementary DNA (cDNA) by High Capacity cDNA Archive Kit (Applied Biosystems, Foster City, CA), and the resulting cDNA was used for real-time PCR analysis using SYBR Green PCR Master Mix in 7300HT Fast Real-Time PCR System (Applied Biosystems). Oligonucleotide primers specific to mouse â-actin and CAR was described in Table S1.

### Motif analyses and transcription factor-binding site over-representation

The core sequence of Antioxidant Response Element (ARE, TGACnnnGC) was searched for in the 5′ region (up to 10 kb upstream of transcription start site) of each gene using Genamics Expression DNA Sequence Analyses Software (GENAMICS, Hamilton, New Zealand). Over-represented conserved transcription factor binding sites in genes induced by Nrf2 activation were searched for by the oPOSSUM system (http://opossum.cisreg.ca/oPOSSUM3). The over-representation was calculated on a conserved region (from 5 kb upstream to 2 kb downstream) around the transcription start site (TSS) of the drug processing genes that were induced by Nrf2, and the over-representation was calculated by comparing with the same region in a battery of drug processing genes that were not altered by Nrf2 as a background.

### Statistical analysis

Data were analyzed using a one-way ANOVA followed by Duncan's multiple range test (p≤0.05) utilizing SigmaStat Software (Systat Software Inc., San Jose, CA). n = 3 for all groups, and values are expressed as mean ± SEM.

## Results

### Uptake transporters

Maximum activation of Nrf2 in Keap1-HKO mice resulted in a marked decrease in mRNA of organic anion-transporting polypeptide 1 (Oatp1a1) and organic anion transporter 2 (Oat2), and a slight decrease in the mRNA of sodium taurocholate cotransporting polypeptide (Ntcp) (Fig. 1). The mRNA of other uptake transporters, namely Oatp1a4, Oatp1b2, organic cation transporter 1 (Oct1), and organic cation/carnitine transporter 2 (Octn2) were not changed with graded Nrf2 activation (Table S2).

### Cytochrome P450 phase-I drug-metabolizing enzymes

Cyp1, Cyp2, and Cyp3 gene families are the major cytochrome P450 enzymes that catalyze phase-I drug metabolism in humans. Compared to wild-type mice, mRNA of Cyp2a5 was 76% lower in Nrf2-null mice, 139% higher in Keap1-KD mice, and 969% higher in Keap1-HKO mice (Fig. 2). The mRNA of Cyp2c50 and Cyp2c54 in Nrf2-null mice was 45 and 65% lower than in wild-type mice, respectively, but in Keap1-KD and Keap1-HKO mice the mRNA of Cyp2c50 and Cyp2c54 tended to be higher than in wild-type mice, although this was not statistically significant. Compared to wild-type mice, the mRNA of Cyp2g1 was 36% lower in Nrf2-null mice, moderately higher with a 91% increase in Keap1-KD mice, and markedly higher with a 850% increase in Keap1-HKO mice. In contrast, Cyp2u1 mRNA decreased with increased Nrf2 activation. More specifically, mRNA of Cyp2u1 was 81% higher in Nrf2-null mice and 48% lower in Keap1-HKO mice. The mRNA of the other 22 genes encoding cytochrome P450 enzymes of the 1–4 families, including Cyp1a2, Cyp2b10, Cyp2c29, Cyp3a11, and Cyp3a13, were not altered with various expression of Nrf2 (Table S3).

### Non-p450 phase-I drug-metabolizing enzymes

**Figure 3:**
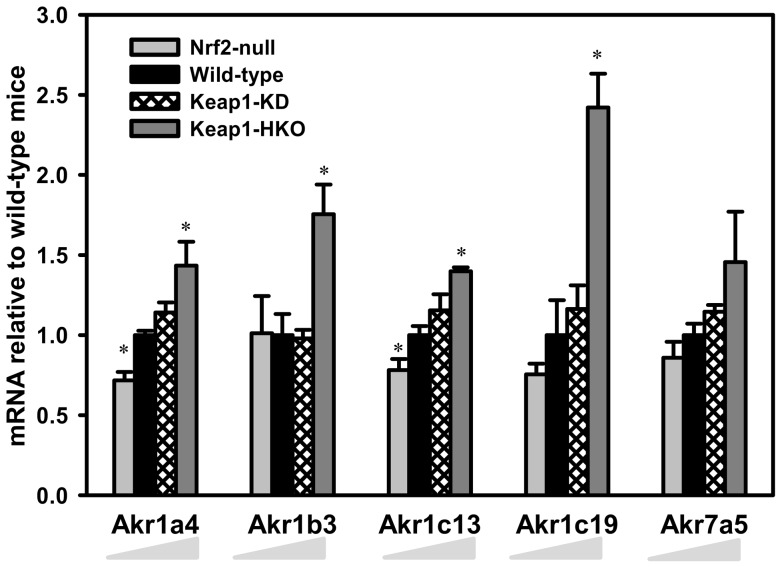
Messenger RNA expression of aldo-keto reductase in a “gene dose-response” model. Data of Nrf2-null, Keap1-KD, and Keap1-HKO mice are normalized by the value of wild-type mice and presented as Mean ± S.E.M. of three mice per group. Asterisks (*) indicate statistically significant differences from wild-type mice (p<0.05).

Aldo-keto reductases (gene name: Akr) catalyze NADPH-dependent reduction of endogenous and xenobiotic substrates. Figure 3 illustrates the mRNA expression of the aldo-keto reductases that were significantly increased in the “gene dose-response” model. Compared to wild-type mice, mRNA of Akr1a4 was 29% lower in Nrf2-null mice, 14% higher in Keap1-KD mice, and 43% higher in Keap1-HKO mice. Messenger RNA of Akr1c13 was 22% lower in Nrf2-null mice and 39% higher in Keap1-HKO mice. Akr1b3 and Akr1c19 mRNA were increased 75 and 142% in Keap1-HKO mice over wild-type mice, respectively. The mRNA of Akr7a5 tended to be lower in Nrf2-null mice, higher in Keap1-KD mice, and highest in Keap1-HKO mice, but these alterations were not statistically significant. Other genes in the Akr gene family, namely Akr1c6, Akr1c12, Akr1c14, Akr1d1, and Akr1e1, were not altered with graded Nrf2 activation (Table S4).

**Figure 4:**
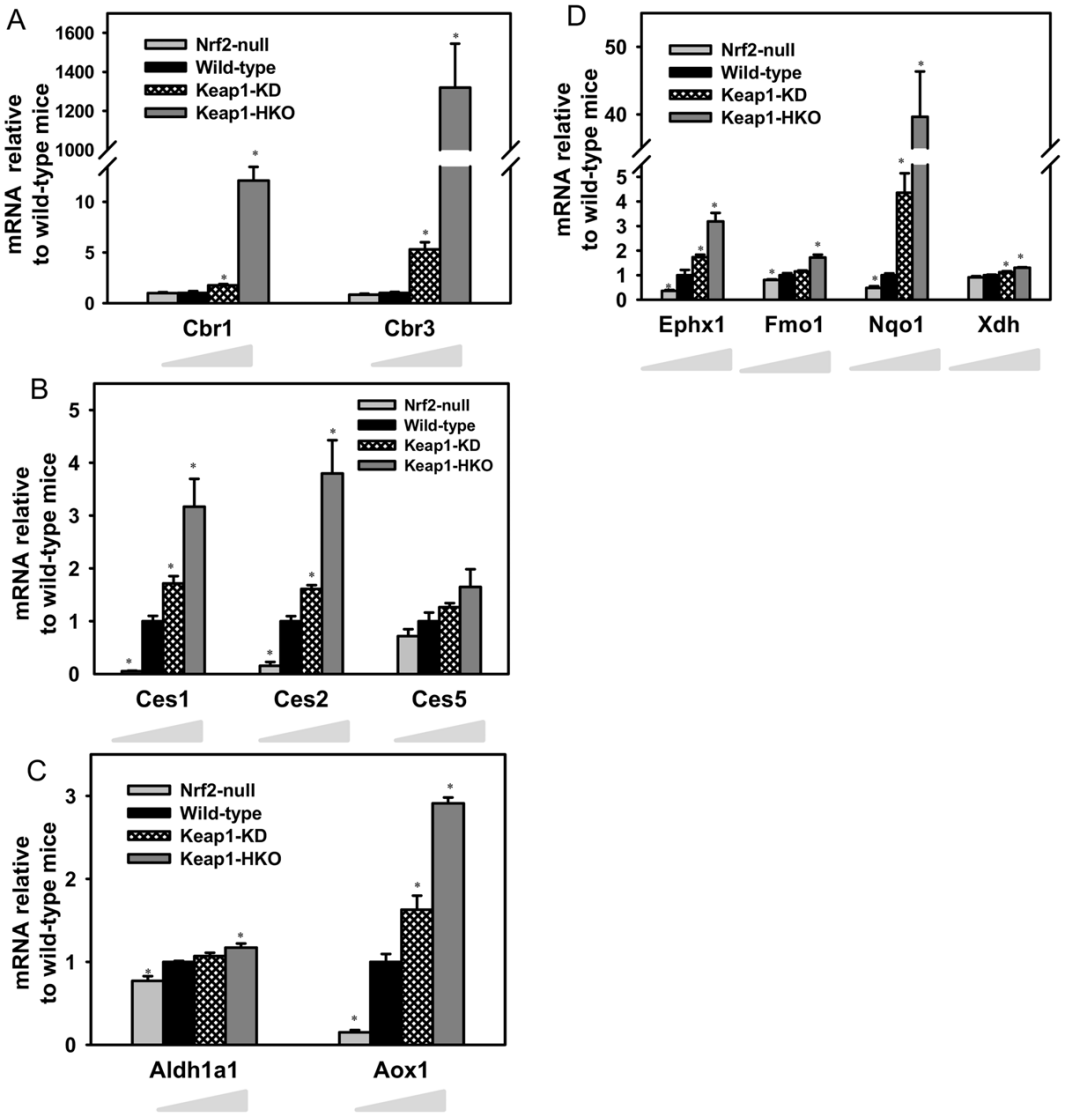
Messenger RNA expression of (A) Carbonyl reductase; (B) Carboxylesterase; (C) Aldehyde dehydrogenase and aldehyde oxidase; (D) Other non-P450 phase-I drug metabolism enzymes in “gene dose-response” model. Data of Nrf2-null, Keap1-KD, and Keap1-HKO mice are normalized by the value of wild-type mice and presented as Mean ± S.E.M. of three mice per group. Asterisks (*) indicate statistically significant differences from wild-type mice (p<0.05).

Carbonyl reductase 1 (Cbr1) was induced in Keap1-KD and Keap1-HKO mice 76 and 1,110%, respectively. Similarly, Cbr3 was induced 430% in Keap1-KD mice and 1318-fold in Keap1-HKO mice (Fig. 4A). Cbr4 mRNA was not changed with graded Nrf2 activation (Table S3). Carboxylesterase 1 (Ces1) and Ces2 mRNAs were lower in Nrf2-null mice, higher in Keap1-KD mice (71 and 61% higher, respectively), and highest in Keap1-HKO mice (216 and 219% higher, respectively). Although Ces5 mRNA tended to be lower in Nrf2-null mice, higher in Keap1-KD mice, and highest in Keap1-HKO mice, these changes were not statistically significant (Fig. 4B). Ces3 and Ces6 mRNA were not changed with graded Nrf2 activation (Table S4).

Of the 13 mRNA of aldehyde dehydrogenases (Aldh1a1, Aldh1a7, Aldh1b1, Aldh1l1, Aldh2, Aldh3a2, Aldh5a1, Aldh6a1, Aldh7a1, Aldh8a1, Aldh9a1, and Aldh16a1) detected in the microarray, only Aldh1a1 mRNA was altered in the “gene dose-response” model, with Nrf2-null lower and Keap1-HKO higher than wild-type mice. The mRNA of aldehyde oxidase (gene name: Aox1) was 85% lower in Nrf2-null mice, and 63% higher in Keap1-KD mice, and 191% higher in Keap1-HKO mice (Fig. 4C). Compared to wild-type mice, mRNA of epoxide hydrolase 1 (Ephx1) was 64% lower in Nrf2-null mice, 73% higher in Keap1-KD mice, and 218% higher in Keap1-HKO mice than wild-type mice. The mRNA of Ephx2 was not changed with graded Nrf2 activation (Table S3). The mRNA of Nqo1 was 52% lower in Nrf2-null mice, 335% higher in Keap1-KD mice, and 3860% higher in Keap1-HKO mice than wild-type mice. The mRNA of Nqo2 was not changed with graded Nrf2 activation (Table S3). The mRNA of Fmo1 and Xdh was slightly increased in Keap1-HKO mice over that in wild-type mice (Fig. 4D), whereas the mRNA of Fmo5 was not altered with graded Nrf2 activation (Table S4).

### Phase-II drug metabolizing enzymes and transporters

**Figure 5:**
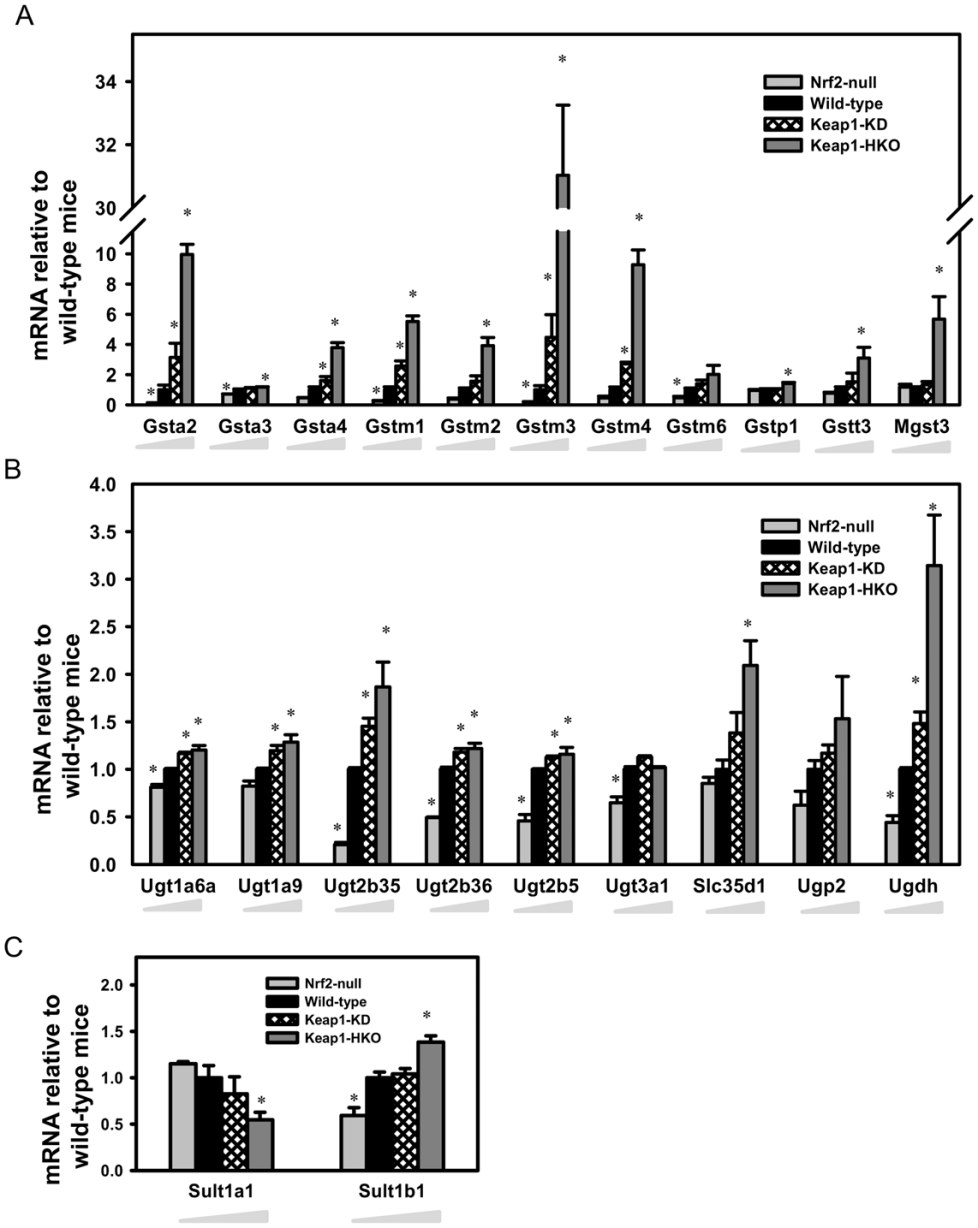
Messenger RNA expression of genes encoding (A) Glutathione *S*-transferase; (B) UDP- glucuronosyltransferase and related enzymes and transproters; (C) Sulfotransferases in a “gene dose-response” model. Data of Nrf2-null, Keap1-KD, and Keap1-HKO mice are normalized by the value of wild-type mice and presented as Mean ± S.E.M. of three mice per group. Asterisks (*) indicate statistically significant differences from wild-type mice (p<0.05).

Of the 25 mouse glutathione *S*-transferases (Gst), 18 Gsts were detected in the microarray, and 11 of them were decreased in the absence of Nrf2, and/or induced with Nrf2 activation (Fig. 5A). The most marked changes in Nrf2-null mice were the 87% decrease in Gsta2, 76% decrease in Gstm1, and 81% decrease in Gstm3. In comparison to wild-type mice, the most marked inductions of Gsts were the 890% increase of Gsta2, 3,000% increase of Gstm3, and 820% increase of Gstm4 in Keap1-HKO mice. Graded Nrf2 activation had no effect on the other 7 Gsts, namely Gstk1, Gstm5, Gstm7, Gsto1, Gstt1, Gstt2, Gstz1 (Table S5).

Graded Nrf2 activation had less of an effect on mRNA profile of UDP-glucuronosyltransferases (Ugt). Of the 21 mouse Ugts, 13 Ugts were detected in the microarray, and 6 were lower without Nrf2, and/or increased with Nrf2 activation (Fig. 5B). The most marked changes in the Ugts in Nrf2-null mice were the 80% lower expression of Ugt2b35, 51% lower Ugt2b36, and 55% lower Ugt2b5 than in the wild-type mice. The most marked increase was 87% of Ugt2b35 in Keap1-HKO mice. UDP-glucose pyrophosphorylase 2 (Ugp2) and UDP-glucose dehydrogenase (Ugdh) are the two enzymes that synthesize UDP-glucuronic acid from glucose. Although Ugp2 mRNA tended to be lower in Nrf2-null mice, higher in Keap1-KD mice, and highest in Keap1-HKO mice, the changes were not statistically significant. In contrast, Ugdh mRNA was significantly lower in Nrf2-null mice (56%), higher in Keap1-KD mice (48%), and highest in Keap1-HKO mice (214%). The solute carrier family 35 member D1 (Slc35d1) is the transporter that imports UDP-glucuronic acid into the endoplasmic reticulum for glucuronidation of substrates. Slc35d1 mRNA was 110% higher in Keap1-HKO mice than in wild-type mice. For the sulfotransferases (Sults), Sult1b1 was higher, whereas Sult1a1 was lower with graded Nrf2 activation (Fig. 5C). Messenger RNA of Sult1d1 and Sult5a1 did not change with graded Nrf2 activation (Table S5).

### Efflux transporters

**Figure 6:**
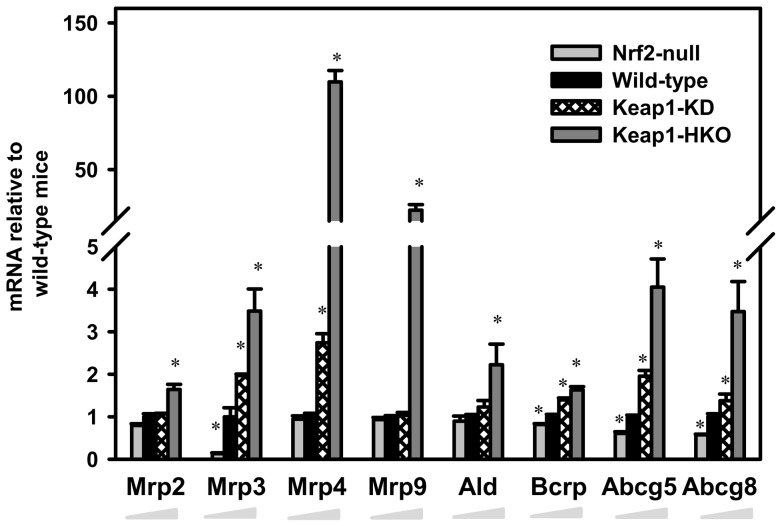
Messenger RNA of efflux transporters in a “gene dose-response” model. Data of Nrf2-null, Keap1-KD, and Keap1-HKO mice are normalized by the value of wild-type mice and presented as Mean ± S.E.M. of three mice per group. Asterisks (*) indicate statistically significant differences from wild-type mice (p<0.05).

For efflux transporters, Nrf2 activation increased 5 out of 6 ATP-binding cassette sub-family C (Mrp, Abcc) members detected by microarray. The most marked changes were the 108- and 21-fold higher Mrp4 and Mrp9 mRNA in Keap1-HKO mice, respectively. The mRNA of Breast cancer resistance protein (Bcrp, Abcg2) was 19% lower in Nrf2-null mice, 40% higher in Keap1-KD mice, and 63% higher in Keap1-HKO mice than in wild-type mice. Similarly, Abcg5 and Abcg8 were constitutively higher with graded Nrf2 activation (Fig. 6). Other efflux transporters, namely Mrp6, multiple drug resistant 1a (Mdr1a/Abcb1a), Mdr2, bile salt export pump (Bsep/Abcb11), and multidrug and toxin extrusion 1 (Mate1/Slc47a1) remained unchanged with graded Nrf2 activation (Table S6).

### Expression of chemical-sensing xenobiotic transcription factors

**Figure 7:**
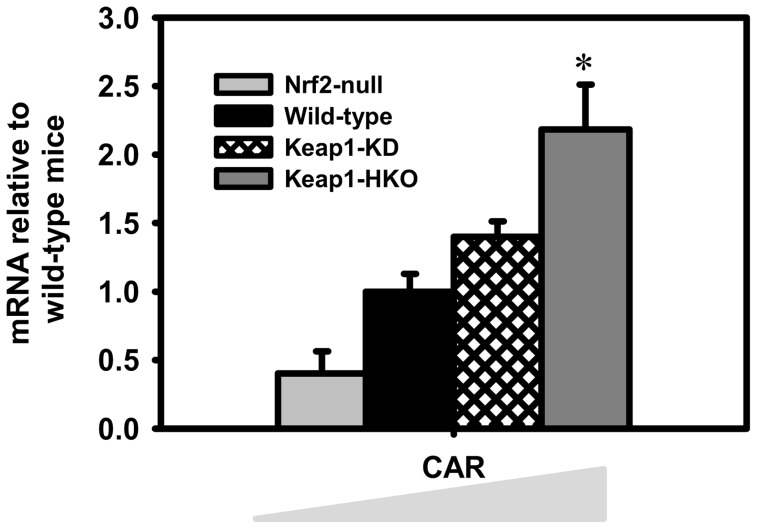
Messenger RNA of CAR in “gene dose-response” model. Data of Nrf2-null, Keap1-KD, and Keap1-HKO mice are normalized by the value of wild-type mice and presented as Mean ± S.E.M. of five mice per group. Asterisks (*) indicate statistically significant differences from wild-type mice (p<0.05).

Compared to wild-type mice, constitutive androstane receptor (CAR, Nr1i3) mRNA was 64% lower in Nrf2-null mice, 41% higher in Keap1-KD mice, and 166% higher in Keap1-HKO mice (Fig. 7). Other genes encoding transcription factors that are involved in drug metabolism, namely pregnane X receptor (PXR, Nr1i2), liver X receptor (LXR, Nr1h3), farnesoid X activated receptor (FXR, Nr1h4), aryl-hydrocarbon receptor (AhR), and hepatic nuclear factor 4, alpha (HNF4α) remained unchanged with graded Nrf2 activation.

### Motif analyses of the 5′ region of drug processing genes

The 5′ region (up to 10kb upstream of TSS) of drug processing genes that are induced (47 genes), not changed (72 genes), and suppressed (5 genes) with graded Nrf2 activation were searched for the core sequence of ARE (TGACnnnGC) as putative Nrf2 binding sites. The gene name, number of AREs found for each gene, and the location of AREs are listed in Table S7, S8, and S9. In the 5′ region up to 2 kb upstream from the TSS, 47 AREs were found in 62% of the genes (29 genes) that were induced with Nrf2 activation, 43 AREs were in 47% of the genes (34 genes) that were not changed with Nrf2 activation, and one ARE was found in 20% of the genes (one gene) suppressed with Nrf2 activation (Table 1). In the 5′ region up to 10 kb upstream from the TSS, 163 AREs were found in 94% of the genes (44 genes) that were induced with Nrf2 activation, 201 AREs were in 86% of the genes (62 genes) that were not changed with Nrf2 activation, and 11 AREs were in the five genes suppressed with Nrf2 activation.

**Table 1:** Summary of total number of AREs in the 5′ region of drug processing genes that were induced, not changed, and suppressed in the Nrf2 “gene dose-response” model.

Gene category	Number of genes	Number of AREs up to 2kb upstream of TSS	Number of gens that contain at least one ARE up to 2kb upstream of TSS	Number of AREs up to 10kb upstream of TSS	Number of gens that contain at least one ARE up to 10kb upstream of TSS
Induced	47	47	29 (62%)	163	44 (94%)
No change	72	43	34 (47%)	201	62 (86%)
Suppressed	5	1	1 (20%)	11	5 (100%)

The core sequence of ARE (TGACnnnGC) was searched for using Genamics Expression DNA Sequence Analyses Software.

### Transcription factor-binding site over-representation

The transcription factor binding site enrichment profile was investigated around the transcription start site of the drug processing genes that were induced by Nrf2. As shown in Table 2, the putative binding site of Nrf2 was greatly enriched in genes that were induced with graded Nrf2 activation. In addition, the putative binding sites for other transcription factors, namely C/EBPα, CTCF, NFATC2, Max, NFkB, USF1, Arnt, PPARγ, and Lhx3, were also enriched in genes that were induced with graded Nrf2 activation.

**Table 2:** Putative binding sites of transcription factors in drug processing genes induced by Nrf2 using the oPOSSUM system.

TF	TF Class	Target gene hits	Background gene hits	Target TFBS nucleotide rate^1^	Background TFBS nucleotide rate^2^	Z-score	Fisher score
Nrf2	Zipper-Type	17	4	0.0134	0.003	37.007	5.173
C/EBPα	Zipper-Type	32	18	0.0431	0.024	21.456	3.129
CTCF	Zinc-coordinating	8	1	0.00489	0.001	19.468	3.432
Nfatc2	Ig-fold	32	22	0.0493	0.031	18.409	1.477
Max	Zipper-Type	17	8	0.0129	0.005	17.872	2.309
Nfkb1	Ig-fold	11	2	0.00495	0.001	17.531	3.94
Usf1	Zipper-Type	21	10	0.0169	0.008	16.307	2.76
Arnt	Zipper-Type	24	11	0.0158	0.008	15.637	3.43
PPARγ::RXRα	Zinc-coordinating	21	8	0.0227	0.014	13.93	3.959
Lhx3	Helix-Turn-Helix	15	9	0.0155	0.009	13.089	1.285

1 : The rate of occurrence of this transcription factor binding sites (TFBS) within the searched regions of the genes induced by Nrf2.

2 : The rate of occurrence of this TFBS within the searched regions of the set of background genes (genes not induced by Nrf2).

## Discussion

**Figure 8:**
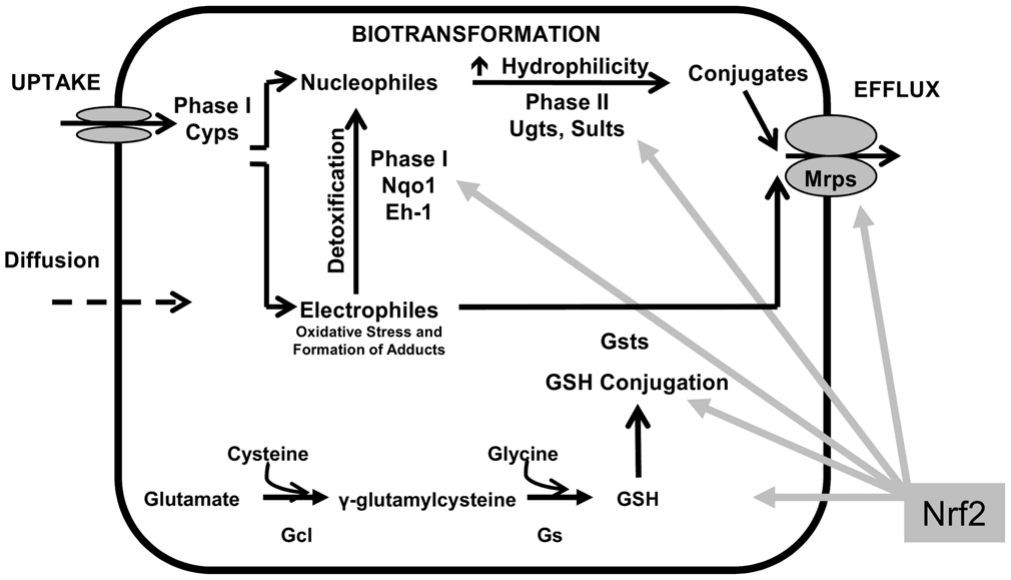
Proposed regulatory model for the role of Nrf2 on drug metabolizing genes in liver. Activation of Nrf2 had a minor effect on uptake transporters and P450 phase-I enzymes that activate xenobiotics into toxicants, markedly induces genes encoding other phase-I enzymes and phase-II enzymes that detoxify toxicants, and also induces efflux transporters that facilitate the elimination of toxicants.

The present data indicate that Nrf2 plays a critical role in regulating mRNA of numerous phase-I and phase-II drug-metabolizing genes as well as a number of efflux transporters that are important for the hepatic disposition of xenobiotics (Fig. 8). Although the role of Nrf2 in regulating some of the drug metabolizing genes were investigated previously through comparing wild-type mice with Nrf2-null or Keap1-HKO mice [19], [20], the present study is among the first reports to make a systematic comparison in four lines of mice with a Nrf2 “gene dose-response”. Taking advantage of microarray analysis, which determines global gene transcription profiles, the present study also systematically compared the importance of Nrf2 in basal and inducible expression of each individual gene involved in drug metabolism and disposition. For example, the present study compared fold-induction of glutathione S-transferase (Gst) gene family by Nrf2 and showed that the mRNA of Gsta3 was increased 20%, whereas the mRNA of Gstm3 was increased over 30-fold in Keap1-HKO mice. In addition, we also report the mRNA of the drug metabolizing genes that are expressed in liver and not changed with absence of Nrf2 or with Nrf2 activation in supplemental tables. For example, although the majority of the Gsts are induced by Nrf2, we report seven Gsts (Gstk1, Gstm5, Gstm7, Gsto1, Gstp1, Gstt1, Gstt2) are not changed with graded Nrf2 activation, indicating that Nrf2 is not qualitatively or quantitatively equally important in induction of this family of enzymes. It is also interesting to note that the mRNA of some genes, such as Fmo1, Xdh, and Aldh1a1, decreased slightly (less than 20%) with the absence of Nrf2, and increased slightly (less than 20%) with maximum Nrf2 activation. However, the mRNA of another battery of genes, including Cyp2a5, Ces1, Ces2, and Gstm3, was almost absent with no Nrf2 in the Nrf2-null mice, and markedly increased (more than 30-fold) with maximum Nrf2 activation in Keap1-HKO mice. This observation suggests that Nrf2 is extremely important in both the basal and inducible expression of these genes.

In contrast to the minor impact of Nrf2 on mRNA of uptake transporters, Nrf2 has a major effet on the abundance of mRNAs of efflux transporters, with most Mrps, Bcrp, and Abcg5/Abcg8 induced in Nrf2 activated mice (Fig. 6). Mrp2 transports GSH and GSH-conjugated xenobiotics from hepatocytes into bile, whereas Mrp3 and Mrp4 transport glucuronated and GSH conjugates, bile acids, and nucleoside analogues from hepatocytes into blood [21], [22]. Bcrp is an efflux transporter that mediates the biliary excretion of sulfate metabolites as well as glucuronated conjugates [23]. Abcg5 and Abcg8 function as a heterodimer and transport cholesterol as well as dietary plant sterols into bile [24]. Taken together, the increase of multiple Mrp isoforms, Bcrp, and Abcg5/Abcg8 probably results in enhanced elimination of xenobiotics from hepatocytes by increased transport into bile and blood.

Nrf2 has a minor role in regulating cytochrome P450s drug metabolizing genes, with only Cyp2a5 and Cyp2g1 induced with Nrf2 activation. The human ortholog of mouse Cyp2a5 is CYP2A6, which is the major CYP that catalyzes the initial metabolism of nicotine, and is also involved in the metabolism of drugs (e.g. valproic acid and pilocarpine) as well as environmental toxicants (e.g. nitrosamines and aflatoxin B1). The present study as well as previous reports show that both human CYP2A6 [25] and mouse Cyp2a5 [26] are induced by Nrf2, indicating Nrf2 may play a role in nicotine metabolism. Cyp2g1 metabolizes exogenous compounds including coumarin and acetaminophen *in vitro* [27], and there is little information about the regulation of Cyp2g1. The present study shows that Cyp2g1 mRNA was lower in the absence of Nrf2 and markedly higher with Nrf2 activation, indicating the novel role of Nrf2 in activating Cyp2g1 transcription.

In contrast to the minor role of Nrf2 in regulating cytochrome P450s, most isoforms of aldo-keto reductases (AKR), including Akr1a4, Akr1b3, Akr1c13, and Akr1c19, were induced with Nrf2 activation (Fig. 3), indicating these genes are novel Nrf2 target genes. Akr1a4 (human ortholog: AKR1A1) plays a key role in ascorbic acid synthesis [28], reduction of trans-muconaldehyde, a cytotoxic metabolite of benzene [29], and scavenges free radicals [30]. The human ortholog of mouse Akr1b3 is AKR1B1, the most extensively studied AKR [31], catalyzes the reduction of a wide range of aldehydes and their glutathione conjugates, including lipid peroxidation products 4-hydroxy-trans-2-nonenal (HNE) and acrolein [32]. Akr1b3 is induced by Nrf2 *in vitro* [33], and human AKR1B10, AKR1C1 and AKR1C2 are induced by Nrf2 in keratinocytes [34]. However, the mRNA of the mouse orthologs of human AKR1B10 (Akr1b10) and AKR1C1 (Akr1c21) remained unchanged with Nrf2 activation (data not shown). This difference may be due to the model used (cell culture versus whole animal), and/or the species (human versus mouse). The AKR7 family catalyzes the reduction of many toxic aldehydes, such as acrolein, methylglyoxal, and especially aflatoxin B1 dialdehyde [35]. Rat Akr7a1 is highly inducible (up to 15-fold) in liver in response to antioxidants [36], whereas mouse Akr7a5 was not induced by antioxidants [37]. Similarly, in the present study, the mRNA of Akr7a5 tended to increase with graded Nrf2 activation, but was not statistically significant (Fig. 2).

In addition to the aldo-keto reductases, the 2 isoforms of carbonyl reductases (Cbr1, Cbr3) were highly inducible by Nrf2. CBR1 is the major enzyme that reduces doxorubicin [38] in humans, and is involved in the detoxification of reactive aldehydes, such as 4-oxonon-2-enal and its GSH conjugate [39]. Human CBR3 catalyzes similar reactions as CBR1 but with narrower substrate specificity, indicating a minor role in xenobiotic metabolism [40]. Keap1-knockdown and then Nrf2 activation resulted in dramatic induction of human CBR3 in cancer cell lines [41]. In parallel with the human cell line study, the present study shows that Cbr3 mRNA was increased more than 1000 fold in livers of Keap1-HKO mice. In addition, Cbr1 was also induced markedly with Nrf2 activation (Fig. 4A), indicating that Nrf2 is an important regulator of Cbr and carbonyl detoxification.

Aldehyde dehydrogenase (Aldh1a1), epoxide hydrolase (Ephx1), and NAD(P)H quinone oxi-reductase (Nqo1) are a group of enzymes that reduce electrophilic substrates into nucleophilic products. Nrf2 is known to be the central regulator that promotes transcription of antioxidant genes. Thus, it is not surprising that Aldh1a1, Eh1, and Nqo1 were induced by Nrf2 in the present study (Fig. 4C and Fig. 4D) and many other studies [42], [43], [44]. Aldehyde oxidase (Aox1) and xanthine dehydrogenase (Xdh) catalyze the generation of reactive oxygen species during oxidation of their substrates, and flavin monooxygenases (Fmo1) catalyze thiobenzamide into an electrophilic intermediate that causes tissue damage [45]. In the present study, Aox1, Xhd, and Fmo1 are induced by Nrf2 (Fig. 4C and Fig. 4D), indicating that whereas most of the enzymes that Nrf2 increases result in decreasing electrophiles and oxidative stress, a few of its target genes can increase electrophiles and oxidative stress.

Nrf2 has a major impact on inducing numerous phase-II drug metabolism genes, with induction of most isoforms of glutathione *S-*transferases, and multiple isoforms of UDP-glucuronosyltransferases. Ugp2 and Ugdh synthesize UDP-glucuronic acid, the substrate for UDP-glucuronosyltransferases catalyzed conjugation, from glucose in the cytosol. Slc35d1 is the transporter that imports UDP-glucuronic acid into the endoplasmic reticulum (ER) and provides the co-substrates for UGT catalyzed conjugation [46]. Nrf2 increases the mRNA of Ugdh and Slc35d1, and tended to increase the mRNA of Ugp2 (Fig. 5B), suggesting that Nrf2 increases the availability of UDP-glucuronic acid in the ER as well as the amount of enzyme for UDP-glucuronic acid conjugation.

In addition to Nrf2, multiple nuclear receptors and other transcription factors, such as AhR, CAR, PXR, PPARα, and FXR, are known to play an important role in regulating the expression of phase-I and -II drug metabolizing enzymes, as well as uptake and efflux transporters [22]. In addition, there are known interactions between Nrf2 and other drug metabolism-related nuclear receptors and transcription factors. For example, a number of AhR target genes are induced by the activation of Nrf2 [47]. The mRNA of Nrf2 is increased in livers of HNF-4α knockout mice [48]. The mRNA and protein of AhR, CAR, and PXR were decreased in livers of Nrf2-null mice [9]. The present study indicates that the mRNA of CAR is almost absent in Nrf2-null mice, and markedly increased in Nrf2 enhanced mice (Fig. 6A), indicating that Nrf2 regulates CAR expression at the mRNA level.

Using *in silico* analyses to search for putative ARE binding sites showed that 44 out of 47 genes induced by Nrf2 in the present study have at least one ARE core sequence (5′-TGACnnnGC-3′) within 10kb upstream from their transcription start sites (Supplement Table 6). The total number of AREs is larger in the set of genes induced by Nrf2 than in the set of genes where mRNAs are not changed or suppressed with Nrf2 activation (Table 1), suggesting that genes are induced in Nrf2 activated mice through direct binding of Nrf2 to promote their transcription. However, it should be noted that 18 out of 47 genes that are induced by Nrf2 do not contain ARE up to 2 kb upstream of the TSS, and three genes (Gsta2, Bcrp, and Abcg8) Nrf2 do not contain ARE up to 2 kb upstream of the TSS. Thus, more experiments (e.g. ChiP-Seq) are needed to confirm the binding of Nrf2 to these putative AREs, and thus whether these genes are direct Nrf2 target genes. In addition to the enrichment of Nrf2 binding sites in the genes responsive to Nrf2 activation, transcription factor over-representation analyses revealed co-existence of ARE and binding sites for other transcription factors, including C/EBPα, NFkB, Arnt:AhR, and PPARγ:RXRα (Table 2), indicating the potential cross-talk between Nrf2 and these transcription-factor signal pathways.

For some of the drug processing genes (example: Nqo1), the mRNA abundance is positively correlated to the enzyme activity [19]. A battery of drug processing genes (example: Cyp2e1) are regulated mainly at post-translational level [49]. However, the mRNA abundance – enzyme activity correlation of most drug processing genes is unknown. Thus, it is not plausible to extrapolate the protein expression or enzyme activity level by comparing mRNA abundance of the genes. Due to the limitations of antibody specificity and availability of *in vivo* enzyme activity assays, the present study only provides the systemic comparison of mRNA abundance of the drug metabolism genes. Western blot and activity assays should be performed to further study the effect of Nrf2 on protein abundance and activity of a specific drug processing enzyme or transporter.

In conclusion, the present study demonstrates that Nrf2, the key transcription factor in protecting against oxidative and electrophilic stress, is also important in regulating hepatic mRNA of phase-I and -II drug metabolizing enzymes as well as uptake and efflux transporters (Fig. 8). Activation of Nrf2 has a minor effect on uptake transporters and P450 phase-I enzymes that activate xenobiotics into toxicants, markedly increased mRNA of other phase-I enzymes and phase-II enzymes that detoxify toxicants, and also increased mRNA of efflux transporters that facilitate the elimination of toxicants.

## Supporting Information

Table S1 **Oligonucleotide sequences for primers specific for mouse β-actin and CAR.**
(DOCX)

Table S2 **List of genes encoding uptake transporters that were not changed with Nrf2 activation.**
(DOCX)

Table S3 **List of cytochrome P450 drug metabolizing genes that were not changed with Nrf2 activation.**
(DOCX)

Table S4 **List of other phase-I drug metabolizing genes that were not changed with Nrf2 activation.**
(DOCX)

Table S5 **List of phase-II drug metabolizing genes that were not changed with Nrf2 activation.**
(DOCX)

Table S6 **List of efflux transporters that were not changed with Nrf2 activation.**
(DOCX)

Table S7 **List of putative AREs at the promoter regions of the drug processing genes induced by Nrf2.**
(DOCX)

Table S8 **List of putative AREs at the promoter regions of the drug processing genes which were not altered with Nrf2 activation.**
(DOCX)

Table S9 **List of putative AREs at the promoter regions of the drug processing genes which were suppressed with Nrf2 activation.**
(DOCX)
