# Supplementary material for: Effect of Graded Nrf2 Activation on Phase-I and -II Drug Metabolizing Enzymes and Transporters in Mouse Liver
Source: PLoS One. 2012 Jul 12;7(7):e39006. doi: 10.1371/journal.pone.0039006 (PMC3395627; doi:10.1371/journal.pone.0039006)
Supplement: Table S1 — Oligonucleotide sequences for primers specific for mouse β-actin and CAR. (DOCX) [file pone.0039006.s001.docx]

**Supplemental table 1**: Oligonucleotide sequences for primers specific for mouse β-actin and CAR.

| **Gene** | **Forward** | **Reverse** |
| --- | --- | --- |
| β-actin | TGACCGAGCGTGGCTACAG | GGGCAACATAGCACAGCTTCT |
| CAR | CTCAAGGAAGCAGGGTCAG | AGTTCCTCGGCCCATATTCT |
