# Supplementary material for: Effect of Graded Nrf2 Activation on Phase-I and -II Drug Metabolizing Enzymes and Transporters in Mouse Liver
Source: PLoS One. 2012 Jul 12;7(7):e39006. doi: 10.1371/journal.pone.0039006 (PMC3395627; doi:10.1371/journal.pone.0039006)
Supplement: Table S2 — List of genes encoding uptake transporters that were not changed with Nrf2 activation. (DOCX) [file pone.0039006.s002.docx]

**Supplemental table 2**: List of genes encoding uptake transporters that were not changed with Nrf2 activation.

| Family | Gene symbol |
| --- | --- |
| Solute carrier organic anion transporter | Oatp1a4 (Slco1a4), Oatp1b2 (Slco1b2) |
| Organic cation transporter | Oct1 (Slc22a1) |
| Organic ation transporter | Oat2 (Slc22a7) |
