# Supplementary material for: Effect of Graded Nrf2 Activation on Phase-I and -II Drug Metabolizing Enzymes and Transporters in Mouse Liver
Source: PLoS One. 2012 Jul 12;7(7):e39006. doi: 10.1371/journal.pone.0039006 (PMC3395627; doi:10.1371/journal.pone.0039006)
Supplement: Table S3 — List of cytochrome P450 drug metabolizing genes that were not changed with Nrf2 activation. (DOCX) [file pone.0039006.s003.docx]

**Supplemental table 3**: List of cytochrome P450 drug metabolizing genes that were not changed with Nrf2 activation.

| Subfamily | Gene symbol |
| --- | --- |
| Cyp1a | Cyp1a2, Cyp1a12 |
| Cyp2b | Cyp2b10 |
| Cyp2c | Cyp2c29, Cyp2c37, Cyp2c44, Cyp2c55, Cyp2c68, Cyp2c70 |
| Cyp2d | Cyp2d9, Cyp2d10, Cyp2d13, Cyp2d22, Cyp2d26 |
| Cyp2e | Cyp2e1 |
| Cyp2f | Cyp2f2 |
| Cyp2j | Cyp2j5, Cyp2j6 |
| Cyp2r | Cyp2r1 |
| Cyp3a | Cyp3a11, Cyp3a13, Cyp3a25 |
