# Supplementary material for: Effect of Graded Nrf2 Activation on Phase-I and -II Drug Metabolizing Enzymes and Transporters in Mouse Liver
Source: PLoS One. 2012 Jul 12;7(7):e39006. doi: 10.1371/journal.pone.0039006 (PMC3395627; doi:10.1371/journal.pone.0039006)
Supplement: Table S4 — List of other phase-I drug metabolizing genes that were not changed with Nrf2 activation. (DOCX) [file pone.0039006.s004.docx]

**Supplemental table 4**: List of other phase-I drug metabolizing genes that were not changed with Nrf2 activation.

| Family | | Gene symbol |
| --- | --- | --- |
|  | Alcohol dehydrogenase | Adh1, Adh4, Adh5, Adh6-psi, Adhfe1 |
|  | Aldehyde dehydrogenase | Aldh1a1, Aldh1a7, Aldh1b1, Aldh1l1, Aldh2, Aldh3a2, Aldh5a1, Aldh6a1, Aldh7a1, Aldh8a1, Aldh9a1, Aldh16a1 |
|  | Aldo-keto reductase | Akr1c12, Akr1c14, Akr1c20, Akr1c6, Akr1d1, Adr1e1, Akr7a5 |
|  | Aldehyde oxidase | Aox3 |
|  | Carbonyl reductase | Cbr4 |
|  | Carboxylesterase | Ces3, Ces6 |
|  | Epoxide hydrolase | Ephx2 |
|  | Flavin containing monooxygenase | Fmo5 |
|  | NAD(P)H quinone dehydrogenase | Nqo2 |
