# Supplementary material for: Effect of Graded Nrf2 Activation on Phase-I and -II Drug Metabolizing Enzymes and Transporters in Mouse Liver
Source: PLoS One. 2012 Jul 12;7(7):e39006. doi: 10.1371/journal.pone.0039006 (PMC3395627; doi:10.1371/journal.pone.0039006)
Supplement: Table S5 — List of phase-II drug metabolizing genes that were not changed with Nrf2 activation. (DOCX) [file pone.0039006.s005.docx]

**Supplemental table 5**: List of phase-II drug metabolizing genes that were not changed with Nrf2 activation.

| Family | Gene symbol |
| --- | --- |
| Glutathione *S*-transferase | Gstk1, Gstm5, Gstm7, Gst01, Gstp1, Gstt1, Gstt2 |
| Sulfotransferase | Sult1a1, Sult1d1, Sult5a1 |
| UDP-glucuronosyltransferase | Ugt2a3, Ugt2b1, Ugt2b34, Ugt2b5, Ugt3a2 |
