# Supplementary material for: Effect of Graded Nrf2 Activation on Phase-I and -II Drug Metabolizing Enzymes and Transporters in Mouse Liver
Source: PLoS One. 2012 Jul 12;7(7):e39006. doi: 10.1371/journal.pone.0039006 (PMC3395627; doi:10.1371/journal.pone.0039006)
Supplement: Table S6 — List of efflux transporters that were not changed with Nrf2 activation. (DOCX) [file pone.0039006.s006.docx]

**Supplemental table 6**: List of efflux transporters that were not changed with Nrf2 activation.

| Family | Gene symbol |
| --- | --- |
| ATP-binding cassette | Abca1, Mdr2 (Abcb4), Bsep (Abcb11), Mrp6 (Abcc6) |
| Solute carrier family | Mate1 (Slc47a1) |
