# Supplementary material for: Effect of Graded Nrf2 Activation on Phase-I and -II Drug Metabolizing Enzymes and Transporters in Mouse Liver
Source: PLoS One. 2012 Jul 12;7(7):e39006. doi: 10.1371/journal.pone.0039006 (PMC3395627; doi:10.1371/journal.pone.0039006)
Supplement: Table S7 — List of putative AREs at the promoter regions of the drug processing genes induced by Nrf2. (DOCX) [file pone.0039006.s007.docx]

**Supplemental table 7**: List of putative AREs at the promoter regions of the drug processing genes induced by Nrf2.

| Gene | Number of AREs | Location (bp from the transcription start site) |
| --- | --- | --- |
| Cyp2a5 | 2 | -2378 ~ -2387 |
|  |  | -4217 ~ -4226 |
| Cyp2c54 | 2 | -765 ~ -774 |
|  |  | -3171 ~ -3180 |
| Cyp2c50 | 4 | -775 ~ -784 |
|  |  | -1020 ~ -1029 |
|  |  | -7871 ~ -7880 |
|  |  | -8961 ~ -8970 |
| Cyp2g1 | 7 | -4165 ~ -4174 |
|  |  | -4736 ~ -4745 |
|  |  | -7444 ~ -7453 |
|  |  | -8565 ~ -8574 |
|  |  | -8581 ~ -8590 |
|  |  | -8597 ~ -8606 |
|  |  | -8613 ~ -8622 |
| Akr1a4 | 2 | -2547 ~ -2556 |
|  |  | -8995 ~ -9004 |
| Akr1c13 | 1 | -7644 ~ -7653 |
| Akr1c19 | 4 | -1450 ~ -1459 |
|  |  | -8694 ~ -8703 |
|  |  | -8805 ~ -8814 |
| Akr7a5 | 8 | -426 ~ -435 |
|  |  | -820 ~ -829 |
|  |  | -2411 ~ -2420 |
|  |  | -7073 ~ -7082 |
|  |  | -7414 ~ -7423 |
|  |  | -7495 ~ -7504 |
|  |  | -9232 ~ -9241 |
| Akr1b3 | 3 | -5841 ~ -5850 |
|  |  | -7714 ~ -7723 |
|  |  | -8807 ~ -8816 |
| Ces1 | 4 | -493 ~ -502 |
|  |  | -1497 ~ -1506 |
|  |  | -2373 ~ -2382 |
|  |  | -2479 ~ -2488 |

**Supplemental table 7-cont’d**: List of putative AREs at the promoter regions of the drug processing genes induced by Nrf2.

| Gene | Number of AREs | Location (bp from the transcription start site) |
| --- | --- | --- |
| Ces2 | 5 | -973 ~ -982 |
|  |  | -1415 ~ -1424 |
|  |  | -3320 ~ -3329 |
|  |  | -9065 ~ -9074 |
|  |  | -9726 ~ -9735 |
| Ces5 | 2 | -4242 ~ -4251 |
|  |  | -4078 ~ 4087 |
| Cbr1 | 4 | -273 ~ -282 |
|  |  | -1758 ~ -1767 |
|  |  | -1804 ~ -1813 |
|  |  | -9400 ~ -9409 |
| Cbr3 | 6 | -3466 ~ -3475 |
|  |  | -4156 ~ -4165 |
|  |  | -6640 ~ -6649 |
|  |  | -8514 ~ -8523 |
|  |  | -8764 ~ -8773 |
|  |  | -9578 ~ -9587 |
| Aox1 | 4 | -309 ~ -318 |
|  |  | -3691 ~ -3700 |
|  |  | -5279 ~ -5288 |
|  |  | -4627 ~ -4636 |
| Aldh1a1 | 3 | -1067 ~ -1076 |
|  |  | -3689 ~ -3698 |
|  |  | -4408 ~ -4417 |
| Ephx1 | 6 | -1761 ~ -1770 |
|  |  | -4027 ~ -4036 |
|  |  | -6393 ~ - 6402 |
|  |  | -8008 ~ -8017 |
|  |  | -8050 ~ -8059 |
|  |  | -9488 ~ - 9497 |
| Nqo1 | 3 | -144 ~ -153 |
|  |  | -2291 ~ -2300 |
|  |  | -3063 ~ -3072 |
| Fmo1 | 1 | -8808 ~ -8817 |
| Xdh | 3 | -1698 ~ -1707 |
|  |  | -4174 ~ -4183 |
|  |  | -6868 ~ -6877 |

**Supplemental table 7-cont’d**: List of putative AREs at the promoter regions of the drug processing genes induced by Nrf2.

| Gene | Number of AREs | Location (bp from the transcription start site) |
| --- | --- | --- |
| Gsta2 | 0 | N/A |
| Gsta3 | 5 | -116 ~ -125 |
|  |  | -544~ - 553 |
|  |  | -1630 ~ -1639 |
|  |  | -9048 ~ -9057 |
|  |  | -9720 ~ -9729 |
| Gsta4 | 5 | -3543 ~ -3552 |
|  |  | -5295 ~ -5304 |
|  |  | -5338 ~ -5347 |
|  |  | -5516 ~ -5525 |
|  |  | -6658 ~ -6667 |
| Gstm1 | 3 | -292 ~ -301 |
|  |  | -1150 ~ -1159 |
|  |  | -7361 ~ -7370 |
| Gstm2 | 2 | -1003 ~ -1012 |
|  |  | -4457 ~ -4466 |
| Gstm3 | 2 | -370 ~ -379 |
|  |  | -1164 ~ -1173 |
|  |  | -1564 ~ -1573 |
|  |  | -7764 ~ -7773 |
| Gstm4 | 6 | -814 ~ -823 |
|  |  | -1192 ~ -1201 |
|  |  | -1891 ~ -1900 |
|  |  | -7017 ~ -7026 |
|  |  | -7084 ~ -7093 |
|  |  | -7085 ~ -7096 |
| Gstm6 | 1 | -306 ~ -315 |
|  |  | -717 ~ -726 |
|  |  | -835 ~ -844 |
|  |  | -2010 ~ -2019 |
|  |  | -4465 ~ - 4474 |
|  |  | -6268 ~ - 6277 |
|  |  | -8141 ~ -8150 |
| Gstp1 | 2 | -1573 ~ -1582 |
|  |  | -5867 ~ -5876 |
| Gstt3 | 1 | -1071 ~ -1080 |
| Mgst3 | 3 | -552 ~ -561 |
|  |  | -9173 ~ -9182 |
|  |  | -9712 ~ -9723 |

**Supplemental table 7-cont’d**: List of putative AREs at the promoter regions of the drug processing genes induced by Nrf2.

| Gene | Number of AREs | Location (bp from the transcription start site) |
| --- | --- | --- |
| Ugt1a6a | 3 | -2295 ~ -2304 |
|  |  | -3144 ~ -3153 |
|  |  | -7971 ~ -7980 |
| Ugt1a9 | 4 | -3866 ~ -3875 |
|  |  | -6125 ~-6134 |
|  |  | -6441 ~-6450 |
|  |  | -8402 ~ -8501 |
| Ugt2b5 | 0 | N/A |
| Ugt2b35 | 2 | -6329 ~ -6338 |
|  |  | -6634 ~ -6643 |
| Ugt2b36 | 1 | -1162 ~ -1171 |
| Ugt3a1 | 4 | -255 ~ -264 |
|  |  | -4085 ~ -4094 |
|  |  | -7745 ~ -7754 |
|  |  | -8203 ~ -8212 |
| Slc35d1 | 4 | -4491 ~ -4500 |
|  |  | -9335 ~ -9344 |
|  |  | -9811 ~ -9820 |
|  |  | -9862 ~ -9871 |
| Ugdh | 3 | -1652 ~ -1661 |
|  |  | -2495 ~ -2504 |
|  |  | -7884 ~ -7893 |
| Ugp2 | 2 | -6936 ~ -6945 |
|  |  | -7639 ~ -7648 |
| Sult1b1 | 0 | N/A |
| Abcc2 | 3 | -89 ~ -98 |
|  |  | -1386 ~ -1395 |
|  |  | -4559 ~ -4568 |
| Abcc3 | 6 | -75 ~ -84 |
|  |  | -564 ~ -573 |
|  |  | -1302 ~ -1311 |
|  |  | -5328 ~ -5337 |
|  |  | -5986 ~ -5995 |
|  |  | -9809 ~ -9818 |
| Abcc4 | 3 | -4415 ~ -4424 |
|  |  | -5986 ~ -5995 |
|  |  | -9790 ~ -9799 |

**Supplemental table 7-cont’d**: List of putative AREs at the promoter regions of the drug processing genes induced by Nrf2.

| Gene | Number of AREs | Location (bp from the transcription start site) |
| --- | --- | --- |
| Abcc9 | 5 | -3334 ~ -3343 |
|  |  | -6273 ~ -6282 |
|  |  | -6657 ~ -6666 |
|  |  | -6943 ~ -6952 |
|  |  | -7117 ~ -7126 |
| Abcd1 | 3 | -1994 ~ -2003 |
|  |  | -4959 ~ -4968 |
|  |  | -5952 ~ -5961 |
| Abcg2 | 0 | N/A |
| Abcg5 | 5 | -461 ~ -470 |
|  |  | -1814 ~ -1823 |
|  |  | -2504 ~ -2513 |
|  |  | -5817 ~ -1826 |
|  |  | -6353 ~ -6362 |
| Abcg8 | 3 | -2308 ~ -2317 |
|  |  | -3229 ~ -3238 |
|  |  | -9488 ~ -9497 |
| Nr1i3 | 1 | -4811 ~ -4820 |
