# Supplementary material for: Effect of Graded Nrf2 Activation on Phase-I and -II Drug Metabolizing Enzymes and Transporters in Mouse Liver
Source: PLoS One. 2012 Jul 12;7(7):e39006. doi: 10.1371/journal.pone.0039006 (PMC3395627; doi:10.1371/journal.pone.0039006)
Supplement: Table S8 — List of putative AREs at the promoter regions of the drug processing genes which were not altered with Nrf2 activation. (DOCX) [file pone.0039006.s008.docx]

**Supplemental table 8**: List of putative AREs at the promoter regions of the drug processing genes which were not altered with Nrf2 activation.

| **Gene** | **Number of AREs** | **Location (bp from the transcription start site)** |
| --- | --- | --- |
| Slco1b2 | 1 | -3821 ~ -3830 |
| Slco1a4 | 3 | -3058 ~ -3067 |
|  |  | -3375 ~ -3384 |
|  |  | -3992 ~ -4001 |
| Slc22a1 | 6 | -112 ~ -121 |
|  |  | -1930 ~ -1939 |
|  |  | -8467 ~ -8476 |
|  |  | -9663 ~ -9672 |
|  |  | -9836 ~ -9845 |
|  |  | -9926 ~ -9935 |
| Cyp1a2 | 3 | -5001 ~ -5010 |
|  |  | -7474 ~ -7483 |
|  |  | -9517 ~ -9526 |
| Cyp2b10 | 2 | -2807 ~ -2816 |
|  |  | -4264 ~ -4273 |
| Cyp2c55 | 3 | -2165 ~ -2174 |
|  |  | -4108 ~ -4117 |
|  |  | -5653 ~ -5662 |
| Cyp2c29 | 2 | -1207 ~ -1216 |
|  |  | -3921 ~ -3930 |
| Cyp2c68 | 3 | -663 ~ -672 |
|  |  | -3474 ~ -3483 |
|  |  | -6598 ~ -6607 |
| Cyp2c37 | 2 | -2664 ~ -2673 |
|  |  | -4548 ~ -4557 |
| Cyp2c70 | 0 |  |
| Cyp2c44 | 3 | -1832 ~ -1841 |
|  |  | -4966 ~ -4975 |
|  |  | -5290 ~ -5299 |
| Cyp2d22 | 1 | -8326 ~ -8335 |
| Cyp2d10 | 2 | -1252 ~ -1261 |
|  |  | -7180 ~ -7189 |
| Cyp2d9 | 4 | -3374 ~ -3383 |
|  |  | -7371 ~ -7380 |
|  |  | -8445 ~ -8454 |
|  |  | -8695 ~ -8704 |
| Cyp2d13- | 2 | -6873 ~ -6882 |
|  |  | -8070 ~ -8079 |
| Cyp2d26 | 3 | -1954 ~ -1963 |
|  |  | -8001 ~ -8010 |
|  |  | -9190 ~ -9199 |

**Supplemental table 8-cont’d**: List of putative AREs at the promoter regions of the drug processing genes which were not altered with Nrf2 activation.

| Gene | Number of AREs | Location (bp from the transcription start site) |
| --- | --- | --- |
| Cyp2e1 | 4 | -2135 ~ -2144 |
|  |  | -2560 ~ -2569 |
|  |  | -6472 ~ -6481 |
|  |  | -8275 ~ -8284 |
| Cyp2f2 | 1 | -6155 ~ -6164 |
| Cyp2j5 | 1 | -723 ~ -732 |
| Cyp2j6 | 2 | -1236 ~ -1245 |
|  |  | -5318 ~ -5327 |
| Cyp2r1 | 1 | -4996 ~ -5005 |
| Cyp3a13 | 1 | -6492 ~ -6501 |
| Cyp3a11 | 6 | -5080 ~ -5089 |
|  |  | -8450 ~ -8459 |
|  |  | -8928 ~ -8937 |
|  |  | -9411 ~ -9420 |
|  |  | -9660 ~ -9669 |
|  |  | -9934 ~ -9943 |
| Cyp3a25 | 2 | -3487 ~ -3496 |
|  |  | -8476 ~ -8485 |
| Adh1 | 1 | -7613 ~ -7622 |
| Adh4 | 2 | -1108 ~ -1117 |
|  |  | -7485 ~ -7494 |
| Adh5 | 1 | -4964 ~ -4973 |
| Adh6-psi | 7 | -3723 ~ -3732 |
|  |  | -4400 ~ -4409 |
|  |  | -5090 ~ -5099 |
|  |  | -5629 ~ -5638 |
|  |  | -8505 ~ -8514 |
|  |  | -8970 ~ -8979 |
|  |  | -9821 ~ -9830 |
| Aldh1a7 | 2 | -1228 ~ -1237 |
|  |  | -5355 ~ -5364 |
| Adhfe1 | 2 | -45 ~ -54 |
|  |  | -1534 ~ -1543 |
| Aldh1b1 | 1 | -9726 ~ -9735 |
| Aldh1l1 | 6 | -723 ~ -732 |
|  |  | -875 ~ -884 |
|  |  | -4132 ~ -4141 |
|  |  | -4372 ~ -4381 |
|  |  | -4450 ~ -4459 |
|  |  | -6611 ~ -6620 |

**Supplemental table 8-cont’d:** List of putative AREs at the promoter regions of the drug processing genes which were not altered with Nrf2 activation.

| Gene | Number of AREs | Location (bp from the transcription start site) |
| --- | --- | --- |
| Aldh2 | 5 | -82 ~ -91 |
|  |  | -4312 ~ -4321 |
|  |  | -5377 ~ -5386 |
|  |  | -6136 ~ -6145 |
|  |  | -9861 ~ -9870 |
| Aldh3a2 | 5 | -415 ~ -424 |
|  |  | -686 ~ -695 |
|  |  | -6506 ~ -6515 |
|  |  | -7240 ~ -7249 |
|  |  | -8823 ~ -8832 |
| Aldh5a1 | 5 | -567 ~ -576 |
|  |  | -1050 ~ -1059 |
|  |  | -5118 ~ -5127 |
|  |  | -7146 ~ -7155 |
|  |  | -8880 ~ -8889 |
| Aldh6a1 | 2 | -5330 ~ -5339 |
|  |  | -9822 ~ -9831 |
| Aldh7a1 | 3 | -78 ~ -87 |
|  |  | -789 ~ -798 |
|  |  | -2977 ~ -2986 |
| Aldh8a1 | 5 | -2705 ~ -2714 |
|  |  | -3391 ~ -3400 |
|  |  | -4101 ~ -4110 |
|  |  | -8256 ~ -8265 |
|  |  | -9053 ~ -9062 |
| Aldh9a1 | 4 | -79 ~ -88 |
|  |  | -4084 ~ -4093 |
|  |  | -8016 ~ -8025 |
|  |  | -8889 ~ -8898 |
| Aldh16a1 | 4 | -2148 ~ -2157 |
|  |  | -3924 ~ -3933 |
|  |  | -4434 ~ -4443 |
|  |  | -7966 ~ -7975 |
| Aox3 | 2 | -5134 ~ -5143 |
|  |  | -7154 ~ -7163 |
| Cbr4 | 4 | -5014 ~ -5023 |
|  |  | -5956 ~ -5965 |
|  |  | -7680 ~ -7689 |
|  |  | -8300 ~ -8309 |

**Supplemental table 8-cont’d**: List of putative AREs at the promoter regions of the drug processing genes which were not altered with Nrf2 activation.

| Gene | Number of AREs | Location (bp from the transcription start site) |
| --- | --- | --- |
| Ces3 | 1 | -7870 ~ -7879 |
| Ces6 | 5 | -2406 ~ -2415 |
|  |  | -2469 ~ -2478 |
|  |  | -2761 ~ -2770 |
|  |  | -4824 ~ -4833 |
|  |  | -9732 ~ -9741 |
| Ephx2 | 2 | -91 ~ -100 |
|  |  | -5661 ~ -5670 |
| Fmo5 | 4 | -624 ~ -633 |
|  |  | -4592 ~ -4601 |
|  |  | -8916 ~ -8925 |
|  |  | -8923 ~ -8932 |
| Nqo2 | 1 | -1185 ~ -1194 |
| Akr1c14 | 2 | -684 ~ -693 |
|  |  | -5363 ~ -5372 |
| Akr1c12 | 3 | -1244 ~ -1253 |
|  |  | -2947 ~ -2956 |
|  |  | -4201 ~ -4210 |
| Akr1c6 | 3 | -6338 ~ -6347 |
|  |  | -9058 ~ -9067 |
|  |  | -9454 ~ -9463 |
| Akr1c20 | 1 | -7630 ~ -7639 |
| Akr1e1 | 1 | -7667 ~ -7676 |
| Akr1d1 | 0 |  |
| Gstk1 | 2 | -3517 ~ -3526 |
|  |  | -8308 ~ -8317 |
| Gstm5 | 6 | -34 ~ -43 |
|  |  | -970 ~ -979 |
|  |  | -3989 ~ -3998 |
|  |  | -6311 ~ -6320 |
|  |  | -8177 ~ -8186 |
|  |  | -9429 ~ -9438 |
| Gstm7 | 7 | -1542 ~ -1551 |
|  |  | -3872 ~ -3881 |
|  |  | -6956 ~ -6965 |
|  |  | -7408 ~ -7417 |
|  |  | -7819 ~ -7828 |
|  |  | -7937 ~ -7946 |
|  |  | -9112 ~ -9121 |

**Supplemental table 8-cont’d**: List of putative AREs at the promoter regions of the drug processing genes which were not altered with Nrf2 activation.

| Gene | Number of AREs | Location (bp from the transcription start site) |
| --- | --- | --- |
| Gsto1 | 4 | -1722 ~ -1731 |
|  |  | -2729 ~ -2738 |
|  |  | -8089 ~ -8098 |
|  |  | -8251 ~ -8260 |
| Gstp1 | 3 | -3365 ~ -3374 |
|  |  | -8351 ~ -8360 |
|  |  | -9234 ~ -9243 |
| Gstt1 | 6 | -375 ~ -384 |
|  |  | -1118 ~ -1127 |
|  |  | -1935 ~ -1944 |
|  |  | -7143 ~ -7152 |
|  |  | -8854 ~ -8863 |
|  |  | -8917 ~ -8926 |
| Gstt2 | 2 | -1425 ~ -1434 |
|  |  | -9592 ~ -9601 |
| Sult1d1 | 3 | -6004 ~ -6013 |
|  |  | -7671 ~ -7680 |
|  |  | -8504 ~ -8513 |
| Sult5a1 | 0 |  |
| Ugt2a3 | 3 | -336 ~ -345 |
|  |  | -7915 ~ -7924 |
|  |  | -9131 ~ -9140 |
| Ugt2b34 | 1 | -982 ~ -991 |
| Ugt2b1 | 4 | -2870 ~ -2879 |
|  |  | -4728 ~ -4737 |
|  |  | -5180 ~ -5189 |
|  |  | -5191 ~ -5200 |
| Ugt2b5 | 3 | -6682 ~ -6691 |
|  |  | -7980 ~ -7989 |
|  |  | -8280 ~ -8289 |
| Ugt3a2 | 5 | -458 ~ -467 |
|  |  | -6168 ~ -6177 |
|  |  | -8687 ~ -8696 |
|  |  | -9299 ~ -9308 |
|  |  | -9770 ~ -9779 |
| Abca1 | 3 | -2456 ~ -2465 |
|  |  | -6460 ~ -6469 |
|  |  | -6577 ~ -6586 |

**Supplemental table 8-cont’d**: List of putative AREs at the promoter regions of the drug processing genes which were not altered with Nrf2 activation.

| Gene | Number of AREs | Location (bp from the transcription start site) |
| --- | --- | --- |
| Abcb4 | 2 | -6164 ~ -6173 |
|  |  | -9242 ~ -8251 |
| Abcb11 | 4 | -1013 ~ 1022 |
|  |  | -2844 ~ -2853 |
|  |  | -6772 ~ -6781 |
|  |  | -7676 ~ -7685 |
| Abcc6 | 2 | -5427 ~ -5436 |
|  |  | -5609 ~ -5618 |
