# Supplementary material for: Effect of Graded Nrf2 Activation on Phase-I and -II Drug Metabolizing Enzymes and Transporters in Mouse Liver
Source: PLoS One. 2012 Jul 12;7(7):e39006. doi: 10.1371/journal.pone.0039006 (PMC3395627; doi:10.1371/journal.pone.0039006)
Supplement: Table S9 — List of putative AREs at the promoter regions of the drug processing genes which were suppressed with Nrf2 activation. (DOCX) [file pone.0039006.s009.docx]

**Supplemental table 9**: List of putative AREs at the promoter regions of the drug processing genes which were suppressed with Nrf2 activation.

| Gene | Number of AREs | Location (bp from the transcription start site) |
| --- | --- | --- |
| Slcoa1a | 2 | -2109 ~ -2118 |
|  |  | -3519 ~ -3528 |
| Slc22a7 | 3 | -3415 ~ -3424 |
|  |  | -5199 ~ -5208 |
|  |  | -9680 ~ -9689 |
| Slc10a1 | 2 | -5497 ~ -5506 |
|  |  | -8439 ~ -8448 |
| Cyp2u1 | 1 | -9927 ~ -9936 |
| Sult1a1 | 3 | -440 ~ -449 |
|  |  | -3473 ~ -3482 |
|  |  | -8969 ~ -8978 |
| Sult1b1 | 0 |  |
